# Supplementary material for: Citrus diseases detection using innovative deep learning approach and Hybrid Meta-Heuristic
Source: PLoS One. 2025 Jan 22;20(1):e0316081. doi: 10.1371/journal.pone.0316081 (PMC11753642; doi:10.1371/journal.pone.0316081)
Supplement: Attachment — Submitted filename: Response to reviewers.pdf [file pone.0316081.s001.pdf]

Response to the Reviewer 1

We are thankful to the reviewer for the encouraging and positive comments to improve the manuscript. We have revised our manuscript based on reviewer 1's comments and highlighted them in the revised manuscript. Please, find below the referees' comments and our responses inserted after each comment in italics.

Review Comments to the Author
Reviewer #1

The study's topic is attractive; however, it requires significant modifications. The authors need to revise specific sections with greater attention.

Author response: *We sincerely thank the reviewer for the encouraging and valuable comments. We are sorry for the mistake caused by our negligence. In the revised manuscript, the mistake has been corrected and highlighted. We have tried our best to address all the concerns and comments and please find our point-by-point response in the following. We hope our amendments are satisfactory and consider our current version of the manuscript for publication.*

Reviewer #1, Concern #1: Lines (47-48), the full names of all models should be provided upon their first mention.

Author response: *Thank you very much for your insightful and constructive feedback. We sincerely apologize for any initial lack of clarity in the manuscript, and we appreciate your careful reading and valuable comments, which have significantly guided our revisions.*

In response to your suggestions, we have clarified the naming and combination of techniques used. In the revised manuscript, we have specified that:

"The proposed model integrates selected features from both deep models, combining them through an array-based method. These features are then classified using a range of supervised learning techniques, including Cost-Sensitive Support Vector Machine (C-SVM), Weighted k-Nearest Neighbors (W-KNN), Quantum Support Vector Machine (Q-SVM), Linear Discriminant Analysis (LDA), Fuzzy k-Nearest Neighbors (F-KNN), k-Nearest Neighbor Bayes (KNB), Multiple Group Support Vector Machine (MG-SVM), Sparse Discriminant Analysis (SDA), Collaborative k-Nearest Neighbors (Co-KNN), and Contextual k-Nearest Neighbors (C-KNN)."

We hope that these revisions address your concerns and enhance the clarity of our study. Thank you again for your invaluable guidance in improving the quality of our work.

Reviewer #1, Concern #2: Lines (35, 37), CV should be written out in full initially, with no need to repeat the Literature Review.

__Author response:__ We apologize once again for any inconvenience caused and for any language that may have inadvertently introduced ambiguity. In response to your valuable suggestions, we have revised the use of CV terminology in the updated version of the manuscript to ensure clarity and precision. Thank you for your guidance in helping us improve the quality of our work.

Reviewer #1, Concern #3: What is the difference between 'CV' mentioned in the introduction and 'convolutional neural networks (CV)' in line 103? Please verify.

__Author response:__ We apologize once again for any inconvenience caused and for any language that may have introduced ambiguity. Following your valuable suggestions, we have revised the manuscript to clarify the use of "CV" terminology. Specifically, we corrected the usage in the Introduction to refer to "Computer Vision (CV)," while in the literature review section, we have replaced "CV" with "convolutional neural networks (CNN)" to maintain accuracy and avoid confusion.

Thank you for your careful attention to detail and for highlighting these points, which have greatly contributed to improving the clarity of our work.

Reviewer #1, Concern #4: Figure 1 needs to be zoomed in to clearly display its details.

__Author response:__ In response to your valuable suggestions, we have adjusted Figure 1 in the revised manuscript to increase its clarity and enhance the visibility of its details. Specifically, we have zoomed in on the figure to ensure that all elements are more easily discernible and contribute to a clearer understanding of the content presented.

Thank you for your careful feedback and for helping us improve the quality of our work.

__**In the revised manuscript, Figure 1.**__

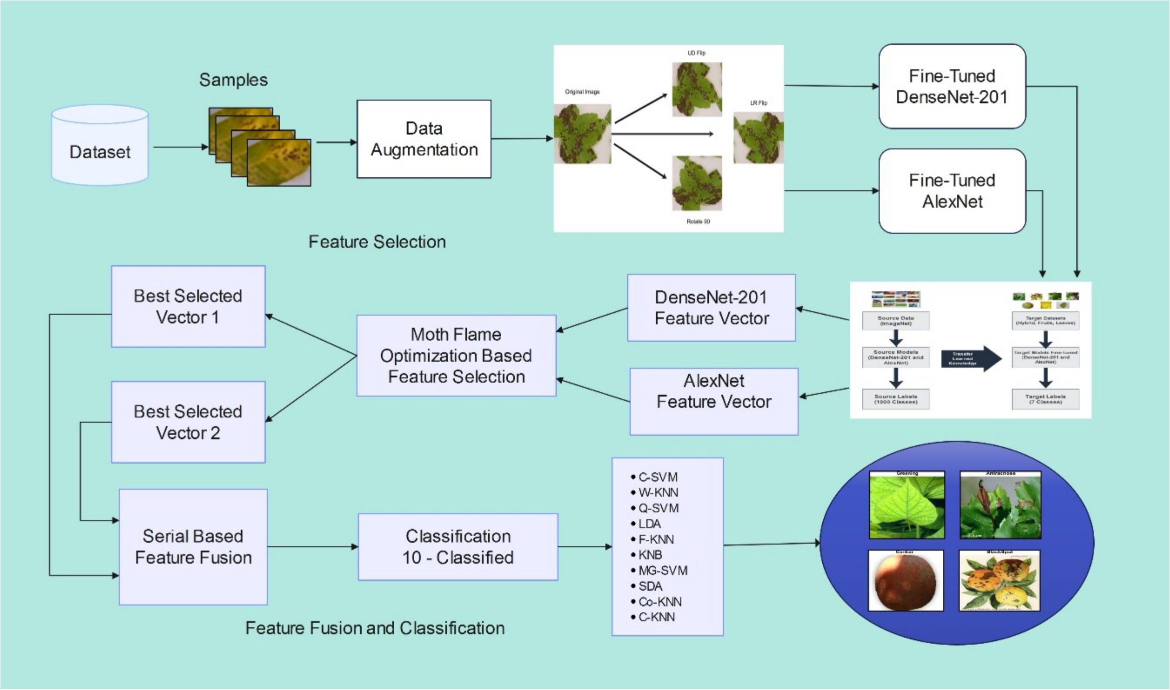

Reviewer #1, Concern #5: Please insert numbering for the manuscript headings, according to the journal guidelines

Proposed Methodology, Data Augmentation, Deep Feature Extraction, Modified DenseNet201…………… etc.

Author response: *Thank you for your kind feedback. We have reviewed the journal guidelines as well as online published articles, and we confirm that heading numbers are not required, as we have used the PLOS ONE LaTeX template for formatting. We appreciate your attention to detail and thank you for your understanding.*

Reviewer #1, Concern #6: Table 1. Procedure for constructing flames in the optimization, and then "Procedure for constructing flames in the optimization:" Do not repeat the rewrite again?

Author response: *We apologize once again for any inconvenience caused and for any language that may have led to ambiguity. In response to your valuable suggestions, we have revised the Table 1 in the updated version of the manuscript as follows:*

Table 1. Procedure for constructing flames in the optimization

**Start with inputs of Moths:**
It uses input data including the positions of moths: X,<br>Fit: the fitness data values of moths,<br>F: the position of the data flame, and<br>$OF(t)$: the fitness values of flames.
**Flame construction in the initial iteration when $t = 1$.**<br>The process involves sorting the Fit vector in ascending order and identifying the sorted indices as $\{j_1, j_2, \ldots, j_N\}$.<br>Construct the flame matrix data $F(t) = \{F_1 \leftarrow X_{j1}, F_2 \leftarrow X_{j2}, \ldots, F_N \leftarrow X_{jN}\}$.
**Flame construction for the rest iteration when $t > 1$.**<br>Create a composite matrix $Dual\_Pop$ by combining matrices $F(t)$ and $X(t-1)$.<br>Form a vector named vector $Dual\_Fit$ by combining matrices $OF(t)$ and $Fit(t-1)$.<br>Sort the vector $Dual\_Fit$ by ascending order data and extract the sorted index in $\{j_1, j_2, \ldots, j_{2N}\}$.<br>Construct the flame matrix $F(t) = \{F_1 \leftarrow X_{j1}, F_2 \leftarrow X_{j2}, \ldots, F_N \leftarrow X_{jN}\}$.

Thank you for your careful review and for helping us improve the clarity and accuracy of our work.

Reviewer #1, Concern #7: The authors mentioned 'Results and Discussions' before 'Experimental Setup,' which is not appropriate. Please move the 'Experimental Setup' section to 'Materials and Methods.'

Author response: Thank you for your thoughtful note. In response to your valuable suggestions, we have moved the "Experimental Setup" section to the "Materials and Methods" section in the updated version of the manuscript to ensure better alignment with standard formatting.

We appreciate your helpful feedback, which has contributed to improving the structure of our work.

Reviewer #1, Concern #8: Table 2, there is no need to repeat the symbols again, such as 'F1-Score' and 'Accuracy'.

Author response: *Thank you for your thoughtful note. In response to your valuable suggestions, we have revised the table in the updated version of the manuscript as follows:*

Table 2. Performance metrics for evaluation

Name	Formula
Sensitivity rate	$\frac{TP}{P} = \frac{\text{Number of true positives}}{\text{Number of true positives+Number of false negatives}}$
Precision rate	$\frac{TP}{TP+FP}$
F1-Score	$2 \times \frac{\text{Precision}\times\text{Recall}}{\text{Precision+Recall}}$
Accuracy	$\frac{TP+TN}{TP+FP+FN+TN}$
True positive rate (TPR)	$\text{TPR} = \frac{\text{True Positive}}{\text{True Positive+False Negative}}$
False positive rate (FPR)	$\text{FPR} = \frac{\text{False Positive}}{\text{False Positive+True Negative}}$

We appreciate your feedback, which has been instrumental in improving the clarity and accuracy of our work.

Reviewer #1, Concern #9: "Results on Hybrid Dataset", revise the heading to ensure it aligns with this section.

Author response: *In response to your valuable suggestions, we have revised the heading "Results on Hybrid Dataset" to "Classification Results with DenseNet-201 and AlexNet on Hybrid Dataset" in the updated version of the manuscript for greater clarity and specificity.*

Thank you for your helpful feedback in enhancing the accuracy of our work.

Reviewer #1, Concern #10: Figures 3-9 have low resolution; please improve it.

Author response: *We sincerely apologize once again for the unclear representation in our previous manuscript. In response to your valuable suggestions, we have revised Figures 3-9 in the updated version of the manuscript. The updated figures are now presented in the best possible resolution, as these images are system-generated.*

Thank you for your constructive feedback, which has greatly contributed to improving the quality of our work.

Reviewer #1, Concern #11: Table 5: Why isn't the same style used as in Tables 6, 7, and 8? Please add % to the first row and remove it from the other fields—there's no need to repeat it. Please fix this issue throughout the manuscript.

Author response: *Yes, you are absolutely right. In response to your valuable suggestions, we have revised Tables 5, 6, 7, and 8 in the updated version of the manuscript to improve clarity and accuracy.*

Thank you for your thoughtful feedback, which has been instrumental in enhancing the quality of our work.

Reviewer #1, Concern #12: The manuscript contains too many confusion matrices. Limit them to two and include the learning curve for the superior model to emphasize the novelty.

Author response: *Yes, you are absolutely right. However, in order to ensure a fair comparison of the applied methods, we believe it is necessary to include all confusion matrices. This will provide a more comprehensive evaluation of the performance of each method.*

Thank you for your valuable input, which has helped us enhance the thoroughness of our analysis.

Reviewer #1, Concern #13: The discussion in the paper is weak; please include more previous studies, according to the lasted studies

- https://doi.org/10.1016/j.dib.2024.110713

- https://doi.org/10.3390/plants13010135

- https://doi.org/10.1007/s11760-024-03452-2

- https://doi.org/10.1016/j.compag.2024.108907

- https://doi.org/10.1016/j.compag.2022.107453

Author response: *In response to your valuable suggestions, we have revised the literature review section by incorporating the article you provided. The updated version of the manuscript now includes the following revision:*

"Traditionally, disease diagnosis in citrus crops [19] has been slow and prone to errors, requiring expert monitoring, which is labor-intensive and costly. In the last decade, image processing and machine learning (ML), particularly deep learning (DL) [20], have been used to automate disease detection in citrus crops. These methods help reduce labor and improve diagnostic accuracy. However, challenges

persist, particularly with image quality being affected by environmental factors and the limitations of conventional imaging devices, which compromise feature extraction accuracy—an essential step in disease detection. Convolutional Neural Networks (CNNs) have shown potential to address these challenges. Models such as InceptionV3, ResNet, and MobileNet have been applied in plant disease detection [21]."

We appreciate your thoughtful feedback, which has significantly improved the clarity and depth of the literature review section.

Reviewer #1, Concern #14: Line (362), Here are the potential areas for future work to…….., Please rewrite this sentence, as it is not ideal to start with 'Here'.

Author response: *In response to your valuable suggestions, we have revised the "Future Work" section in the updated version of the manuscript as follows:*

"In the future, this research will expand the scope to include additional datasets with diverse and complex images encompassing various diseases. Moreover, it will focus on improving the computational efficiency of the model for real-time use in field conditions, exploring model compression techniques, and enhancing generalization by incorporating diverse environmental data to address potential dataset biases."

Thank you for your insightful feedback, which has helped us refine the direction of our future research.

Reviewer #1, Concern #15: This paper requires significant revisions before it can be considered complete. Therefore, I recommend a major revision. Thank you.

Author response: *Thank you for your thoughtful and constructive feedback, which has been invaluable in refining our manuscript. We believe our findings will be of significant interest to the community, and we have meticulously addressed your comments, making revisions to enhance both the clarity and quality of our work.*

We respectfully submit the revised version for your consideration for publication in ***PLOS ONE****. We sincerely appreciate your time and dedication in reviewing our paper and welcome any further feedback or suggestions you may have. Please do not hesitate to reach out with any additional questions or recommendations.*

Once again, thank you for your valuable insights and guidance.

Response to the Reviewer 2

We are thankful to the reviewer for the encouraging and positive comments to improve the manuscript. We have revised our manuscript based on reviewer 2's comments and highlighted them in the revised manuscript. Please, find below the referees' comments and our responses inserted after each comment in italics.

Review Comments to the Author
Reviewer #2

A major revision is being suggested. Following are the point-to-point comments that need to be addressed for further review:

Author response: *We are sincerely thankful to the reviewer for the encouraging and valuable comments. We are sorry for the mistake caused by our negligence. In the revised manuscript, the mistake has been corrected and highlighted. We have tried our best to address all the concerns and comments and please find our point-by-point response in the following. We hope that our amendments are satisfactory and consider our current version of the manuscript for publication.*

Reviewer #2, Concern #1: Abstract: The motivation of the research is not clear. It is suggested that the authors include a clear motivation statement in the abstract. Also, one sentence presenting the major findings with quantitative support needs to be included as the second sentence from the end of the abstract.

Author response: *The authors are deeply grateful for your efforts and insightful comments. We apologize for any inconvenience caused by the previous ambiguity and inappropriate language. The manuscript has been extensively revised to clarify these points.*

In response to your valuable suggestions, we have revised the abstract in the updated version of the manuscript as follows:

Abstract

Citrus farming is one of the major agricultural sectors of Pakistan and currently represents almost 30% of total fruit production, with its highest concentration in Punjab. Although economically important, citrus crops like sweet orange, grapefruit, lemon, and mandarins face various diseases like canker, scab, and black spot, which lower fruit quality and yield. Traditional manual disease diagnosis is not only slow, less accurate, and expensive but also relies heavily on expert intervention. To address these issues, this research examines the implementation of an automated disease classification system using deep learning and optimal feature selection. The system incorporates data augmentation and transfer learning with pre-trained models such as DenseNet-201 and AlexNet to improve diagnostic accuracy, efficiency, and cost-effectiveness. Experimental results on a citrus leaves dataset show an impressive 99.6% classification accuracy. The proposed framework outperforms existing methods, offering a robust and scalable solution for disease detection in citrus farming, contributing to more sustainable agricultural practices.

In addition, we have clarified the statement of motivation to emphasize the significance of citrus farming as a major cash crop in Pakistan, as well as the crop's susceptibility to diseases, which are key reasons for conducting this study.

Main Results: The research achieves 99.6% accuracy, surpassing current state-of-the-art methods. This key finding highlights the potential applications of the system, demonstrating its greater efficiency in disease detection and management.

Thank you for your thoughtful feedback, which has contributed significantly to enhancing the clarity and impact of our work.

Reviewer #2, Concern #2: Introduction: This is written very poorly. The authors are suggested to merge the Literature review with the introduction to correctly identify the research gap and formulate the objectives in line with the research gap.

Author response: *We sincerely apologize for any previous inconvenience and for any inappropriate language that may have caused ambiguity. In response to your valuable suggestions, we have revised the objectives and research gap in the updated version of the manuscript as follows:*

In the Introduction Section:

"Research Objectives
The primary contributions of this study are as follows:
- Deep features are generated for each model, which are subsequently refined using the Moth-Flame Optimization Algorithm.
- We enhanced the pre-trained DenseNet-201 and AlexNet models, which were further trained using deep transfer learning.
- The proposed model combines the selected features from both deep models using an array-based method and classifies them using various supervised learning approaches, including Cost-Sensitive Support Vector Machine (C-SVM), Weighted k-Nearest Neighbors (W-KNN), Quantum Support Vector Machine (Q-SVM), Linear Discriminant Analysis (LDA), Fuzzy k-Nearest Neighbors (F-KNN), k-Nearest Neighbor Bayes (KNB), Multiple Group Support Vector Machine (MG-SVM), Sparse Discriminant Analysis (SDA), Collaborative k-Nearest Neighbors (Co-KNN), and Contextual k-Nearest Neighbors (C-KNN).
- The proposed framework is examined at each phase and compared to recent techniques, affirming its superior performance and robustness in the detection of citrus diseases."

In the Literature Review Section:

"Research Gap
Despite the availability of deep learning methods for citrus disease detection, a gap exists in optimizing feature extraction and selection, which is critical for improving classification accuracy. Most existing approaches fail to enhance the extracted features or incorporate effective feature selection algorithms. Furthermore, they often rely on a single model, which may not effectively handle the diverse range of

citrus diseases. This paper addresses this gap by proposing a hybrid deep learning framework that combines DenseNet-201 and AlexNet with the Moth-Flame Optimization Algorithm (MFO) for optimal feature selection. By leveraging deep transfer learning and a feature fusion technique, this study aims to improve classification accuracy and robustness in citrus disease detection."

Thank you for your constructive feedback, which has been instrumental in refining the manuscript and enhancing its clarity.

Reviewer #2, Concern #3: Line 130: The authors have mentioned that this study utilizes three datasets. However, no details related to the data set are provided. Also, the author has not declared that the dataset is public or private, developed by authors, or accessed from other sources. The details must be stated, and appropriate citations must be provided.

Author response: *In response to your valuable suggestions, we have revised the Datasets section in the updated version of the manuscript as follows:*

Dataset
The experiments in this study were carried out using a publicly available citrus disease dataset. The dataset consists of 7,500 images depicting seven classes of citrus fruit and leaf diseases, including Anthracnose, citrus greening, black spot, canker, melanose, citrus scab, and healthy citrus plants. The dataset was created by combining three different sources: the Hybrid Dataset, the Fruits Dataset, and the Leaves Dataset.

- Hybrid Dataset: Contains 3,988 images, including both fruit and leaf symptoms of various citrus diseases.
- Fruits Dataset: Includes 1,328 images of diseased and healthy citrus fruits.
- Leaves Dataset: Contains 2,184 images of citrus leaves showing different diseases.

Additionally, we have included the Data Availability Statement:

Data Availability Statement
The dataset is available at: https://www.kaggle.com/datasets/myprojectdictionary/citrus-leaf-disease-image

We appreciate your insightful feedback, which has helped to clarify the dataset description and enhance the transparency of our research.

Reviewer #2, Concern #4: Figure 3, Figure 6, figure 7, figure 8, figure 9: The quality of the figure must be enhanced. The current figure quality is blurry and difficult to read.

Author response: *We sincerely apologize once again for any inconvenience caused and for any language that may have led to ambiguity. In response to your valuable suggestions, we have revised*

Figures 3-9 in the updated version of the manuscript. The updated figures are now presented in the best possible resolution, as these images are system-generated.

Thank you for your constructive feedback, which has significantly contributed to improving the clarity and quality of our work.

Reviewer #2, Concern #5: Figure 10, Figure 11, and Figure 12 present the same quantitative values throughout the graph. Hence, it is suggested that the authors remove the table below and make good-quality plots for better representation and understanding.

Author response: *In response to your valuable suggestions, we have revised the figures by removing the table below them in the updated version of the manuscript. This adjustment was made to improve clarity and streamline the presentation of the data.*

Thank you for your thoughtful feedback, which has helped enhance the overall structure and presentation of our manuscript.

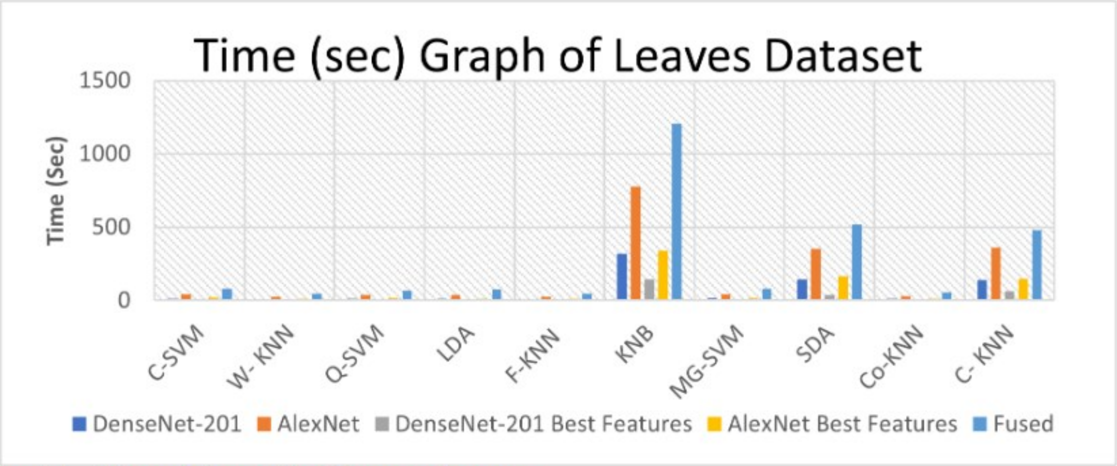

Fig 11. Time (s) graph of leaves dataset.

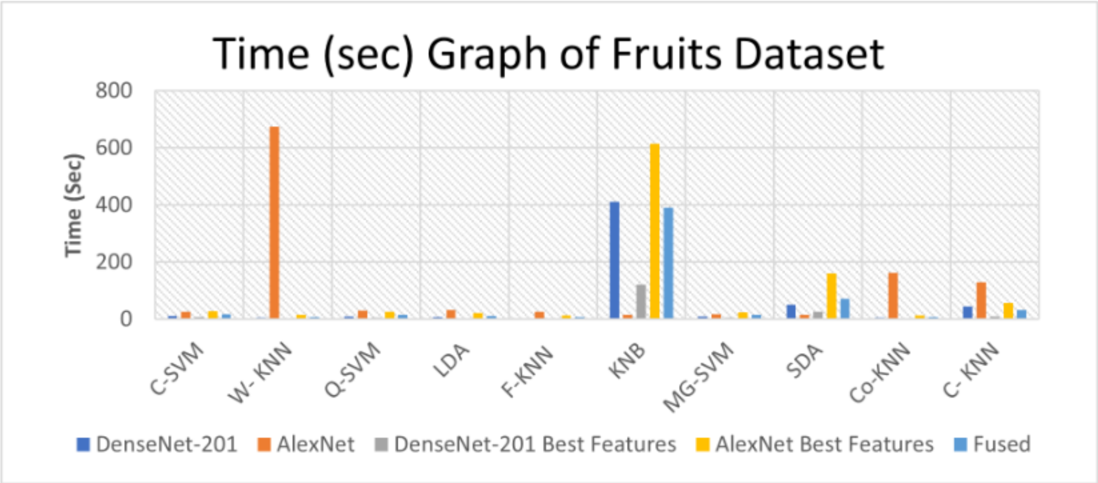

Fig 12. Time(s) graph of fruits dataset.

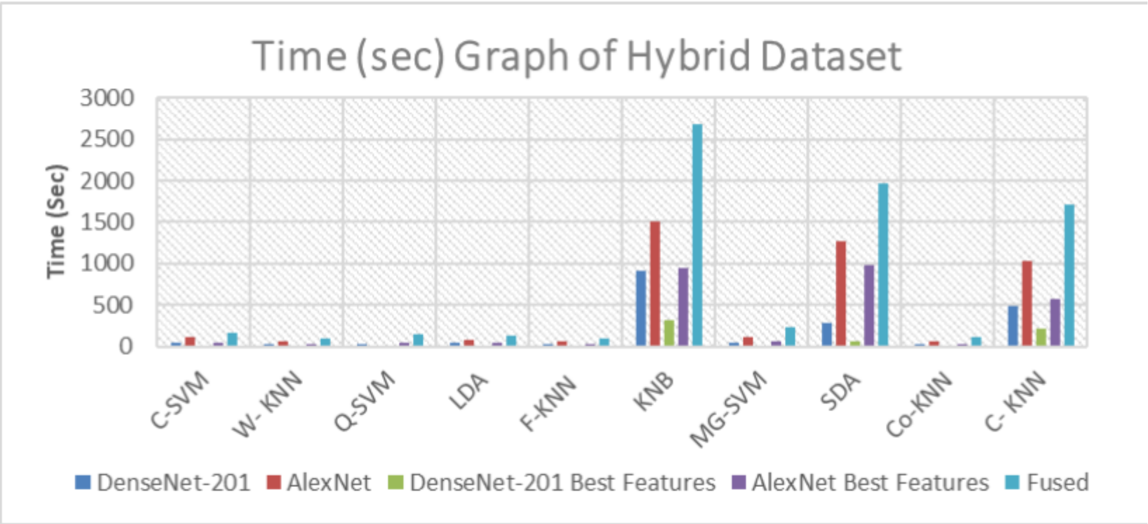

Fig 10. Time (s) graph of hybrid dataset.

Reviewer #2, Concern #6: Line 319 to 347, the Authors discussed the obtained results, and one table has been appended below with the title State of the art comparisons. However, the critical comparison and reasoning behind the obtained results are missing in the discussion section. The authors are expected to discuss the obtained results and compare them with the existing results to explain the reasoning behind the presented phenomenon. Hence, it was suggested that the discussion be enhanced as suggested.

Author response: *In response to your valuable suggestions, we have enhanced the Discussion and State-of-the-Art Comparisons sections in the updated version of the manuscript as follows:*

"Discussions
This section offers a comprehensive discussion of the proposed framework, presenting results across Tables 3 to 10. The experiments encompass three datasets, with five experiments conducted for each dataset. The results for the hybrid dataset are found in Tables 3, 4, 5, and 6. They reveal the highest accuracy values to be 99.5\%, 97.36\%, 99.4\%, and 96.9\%, respectively. The accuracy analysis shows that applying the feature selection method to DenseNet-201 features slightly decreases accuracy from 99.5\% to 99.4\%. Likewise, for AlexNet features, the accuracy decreases from 97.36\% to 96.9\% when the feature selection method is applied. A fusion step is executed, and the results are presented in Table 7. Notably, the accuracy improves, but there is an increase in computational time.

In the leaves dataset, the results in Tables 8, 9, and 10 indicate the highest accuracy values of 98.9\%, 94.3\%, 98.7\%, and 94.1\%, respectively. These accuracy results reveal that when the feature selection method was applied to DenseNet-201 features, the accuracy dropped from 98.9\% to 98.7\%. Similarly, for AlexNet features, the accuracy decreased from 94.3\% to 94.1\%. Feature fusion is executed, and the results are documented in Table 12. Notably, the accuracy has improved, but the computational time has increased.

The application of feature selection and fusion methods, combined with deep learning models for citrus disease identification, has shown considerable improvements over individual classifiers proposed in the

literature. Experiments were conducted on three datasets—Hybrid, Leaves, and Fruit—with five experiments on each. This section critically analyzes the results, compares them with existing literature, and discusses their implications for future research.

Challenges and future research directions increased computational time after feature fusion, especially in the Hybrid and Leaves datasets, highlights the challenge of deploying deep learning models for real-time agricultural applications. Future work should focus on optimizing computational efficiency through techniques like model pruning, quantization, or distillation to reduce the complexity of fused models without sacrificing much accuracy, enabling deployment on resource-constrained devices like mobile phones."

"State of the art comparisons
A comparison of the results of the proposed framework to the recent state-of-the-art techniques is given in Table \ref{tabsota}. Comparison with State-of-the-Art Methods compares the proposed framework to existing methods. Our approach demonstrated superior performance, achieving 99.9\% and 99.6\% accuracies with DenseNet-201 for the Hybrid and Fruit datasets."

We appreciate your thoughtful suggestions, which have significantly improved the clarity and depth of these sections.

Reviewer #2, Concern #7: The conclusions must be proper and in alignment with the objectives. Also, it is expected that authors must present major findings with quantitative support. The authors obtained multiple parameters but only presented parameter accuracy and provided it for the three datasets. No proper and critical write-up is expected here. Hence, the authors are advised to rewrite the conclusion in a maximum of 200 words and include 1-2 sentences at the end that relate to the future direction of the work.

Author response: *In response to your valuable suggestions, we have rewritten the Conclusion section in the updated version of the manuscript as follows:*

"Conclusion and future direction
This study investigates deep learning and has made remarkable strides in agriculture, notably in its ability to automate the identification of plant diseases while reducing the need for extensive human involvement. It aimed to create an automated system for identifying diseases in Citrus fruits and leaves using deep learning approaches and best-feature selection. Data augmentation is performed early to expand the dataset and enhance the robustness of deep learning models. Subsequently, two pre-trained models were adapted and fine-tuned using transfer learning on the augmented datasets. Features were extracted through a proposed MFO method and combined using a serial-based approach. The resulting features were then classified using various supervised learning algorithms. The experimental process covered three distinct datasets: Hybrid dataset, Leaves dataset, and Fruits dataset, achieving high accuracies of 99.9\%, 99.6\%, and 99.1\%, respectively. This study showcases the efficacy of deep learning and feature selection in automating the identification of citrus fruit and leaf diseases with remarkable accuracy. In the future, this research expand the scope to include additional datasets with diverse and complex images encompassing various diseases. Moreover, it should also focus on

improving the computational efficiency of the model for real-time use in field conditions, exploring model compression techniques, and enhancing generalization by incorporating diverse environmental data to address potential dataset biases."

Reviewer #2, Concern #8: References: The authors are advised to include more recent references, particularly from journals such as Elsevier, Springer, PLOS ONE, etc. Currently, no articles from 2024 have been cited. Only two articles from 2023 and one from 2022 are referenced. This suggests either an inadequate literature survey or an outdated research domain. It is strongly recommended that the authors incorporate recent citations to ensure the study's relevance.

Author response: *In response to your valuable suggestions, we have added the latest references from 2024 in the updated version of the manuscript. These references have been carefully selected to ensure the inclusion of recent advancements in the field and to provide a more comprehensive background for our research.*
We appreciate your recommendation to incorporate up-to-date sources, as it has strengthened the manuscript and ensured its relevance to the current state of the art.
Thank you again for your constructive feedback, which has greatly contributed to enhancing the quality of our work.

"32. Abbas MA, Munir K, Raza A, Amjad M, Samee NA, Jamjoom MM, Ullah Z. A novel meta learning based stacked approach for diagnosis of thyroid syndrome. PloS One 2024; 19(11): e0312313.
33. Akhtar I, Atiq S, Shahid MU, Raza A, Samee NA, Alabdulhafith M. Novel glassbox based explainable boosting machine for fault detection in electrical power transmission system. PloS One 2024; 19(8): e0309459.
34. Tanveer MU, Munir K, Raza A, Almutairi MS. Novel artificial intelligence assisted Landsat-8 imagery analysis for mango orchard detection and area mapping. PloS One 2024; 19(6): e0304450."

Author's final remark: *Thank you for your thoughtful and constructive feedback, which has been invaluable in refining our manuscript. We believe our findings will be of significant interest to the community, and we have meticulously addressed your comments, making revisions to enhance both the clarity and quality of our work.*

*We respectfully submit the revised version for your consideration for publication in **PLOS ONE**. We sincerely appreciate your time and dedication in reviewing our paper and welcome any further feedback or suggestions you may have. Please do not hesitate to reach out with any additional questions or recommendations.*

Once again, thank you for your valuable insights and guidance.
